# First Ecological Study of the Bawean Warty Pig (*Sus blouchi*), One of the Rarest Pigs on Earth

**DOI:** 10.1371/journal.pone.0151732

**Published:** 2016-04-06

**Authors:** Mark Rademaker, Erik Meijaard, Gono Semiadi, Simen Blokland, Eric W. Neilson, Eva Johanna Rode-Margono

**Affiliations:** 1 Department of Animal Management, VHL University of Applied Sciences, Leeuwarden, The Netherlands; 2 Bawean Endemics Conservation Initiative, Jakarta, Indonesia; 3 IUCN/SSC Wild Pig Specialist Group, Jakarta, Indonesia; 4 ARC Centre of Excellence for Environmental Decisions (CEED), The University of Queensland, Brisbane, QLD4072, Australia; 5 Research Centre for Biology, Zoology Division, The Indonesian Institute of Sciences, Cibinong, Indonesia; 6 Department of Biological Sciences, University of Alberta, Edmonton, Canada; 7 The North of England Zoological Society / Chester Zoo, Chester, United Kingdom; Fenner School of Environment and Society, AUSTRALIA

## Abstract

The Bawean warty pig (*Sus blouchi*) is an endemic pig species confined to the 192 km^2^ large island of Bawean in the Java Sea, Indonesia. Due to a lack of quantitative ecological research, understanding of natural history and conservation requirements have so far been based solely on anecdotal information from interviews with local people and study of captive and museum specimens. In this study we provide the first assessment of population and habitat preferences for *S*. *blouchi* by using camera trapping. From the 4th of November 2014 to January 8th 2015, we placed camera traps at 100 locations in the forested protected areas on Bawean. In 690.31 camera days (16567.45 hours) we captured 92 independent videos showing *S*. *blouchi*. Variation in *S*. *blouchi* trapping rates with cumulative trap effort stabilized after 500 camera days. An important outcome is that, in contrast to the suggestion of previous assessments, only *S*. *blouchi* was detected and no *S*. *scrofa* was found, which excludes hybridization threats. We fitted a Random Encounter Model, which does not require the identification of individual animals, to our camera-trapping data and estimated 172–377 individuals to be present on the island. Activity patterns and habitat data indicate that *S*. *blouchi* is mainly nocturnal and prefers community forests and areas near forest borders. Next to this, we found a positive relationship between *S*. *blouchi* occupancy, distance to nearest border, litter depth and tree density in the highest ranking occupancy models. Although these relationships proved non-significant based on model averaging, their presence in the top ranking models suggests that these covariables do play a role in predicting *S*. *blouchi* occurrence on Bawean. The estimated amount of sites occupied reached 58%. Based on our results, especially the estimation of the population size and area of occupancy, we determine that the species is Endangered according to the IUCN/SSC Red List criteria.

## Introduction

In Southeast Asia (SEA) sea level fluctuations since the early Pliocene created frequent island connections and isolations, which provided ample opportunities for speciation to occur [1, 2]. This is one reason why the area hosts the highest wild pig (family Suidae) diversity in the world [3]. The exact number of species and subspecies of wild pigs in SEA remains an ongoing taxonomic debate [4–6]. The International Union for Conservation of Nature (IUCN) currently acknowledges nine species within the genus *Sus*, six of which are considered as threatened, while the three species in the genus *Babyrousa*, the other Suidae in SEA, are also threathened [7].

Recognizing the threats faced by many of these species, the IUCN/SSC Wild Pig Specialist Group (WPSG) organized a workshop on SEA wild pig species in November 2013. The aim was to develop immediate action points for improved conservation of threatened wild pig taxa in Asia [8]. A taxon that was extensively discussed is the Bawean warty pig (*Sus blouchi*), an island endemic confined to Bawean Island in the Java Sea [8, 9]. Based on morphological research, it was recently proposed to upgrade the species from a subspecies of the Javan warty pig (*S*. *verrucosus*) to full species level [5].

For both *S*. *blouchi* and *S*. *verrucosus*, quantitative ecological research is almost entirely missing. Population assessments and species descriptions have been based on data obtained through interviews with local people, study of historic literature and through observation of captive animals or museum specimens [5, 9–12]. This has allowed an IUCN Red List classification of *S*. *verrucosus* as Endangered [13], which currently also covers *S*. *(v*.*) blouchi*, because the latter taxon has not yet been assessed separately. The area of Bawean Island, however, is less than 200 km^2^, compared to the 139,000 km^2^ of Java, suggesting that one threat assessment for both taxa is likely to be inaccurate. A separate Red List assessment of *S*. *blouchi* was attempted during the November 2013 workshop, but participants concluded that insufficient information on range, population size and threats was available to determine its conservation status [8]. To assist the development of appropriate conservation measures and the Red Listing of *S*. *blouchi* we provide the first quantitative population estimate and habitat preferences based on camera trapping, as well as a summary of threats and habitat quality based on recent surveys on Bawean. We also assess whether *S*. *blouchi* is the only suid species on Bawean or whether *S*. *scrofa* also occurs as suggested by previous surveys [10, 14], the latter suggesting that hybridization between the two species would be an additional threat to *S*. *blouchi*.

## Materials and Methods

### Ethics statement

Data collection used non-invasive, remotely set camera traps and did not involve contact or direct interaction with animals. Fieldwork was performed under research permit number 367/SIP/FRP/SM/X/2014 to MR, issued by The Indonesian Ministry for Research and Technology (RISTEK). A permit to enter and work in strict nature reserves and wildlife reserves was obtained from the Office of Conservation of Natural Resources (BBKSDA), part of the Ministry of Forestry, under SIMAKSI no. SI.21/BBKSDA.JAT-2.1/ 2014 to MR.

### Study site

**Fig 1:**
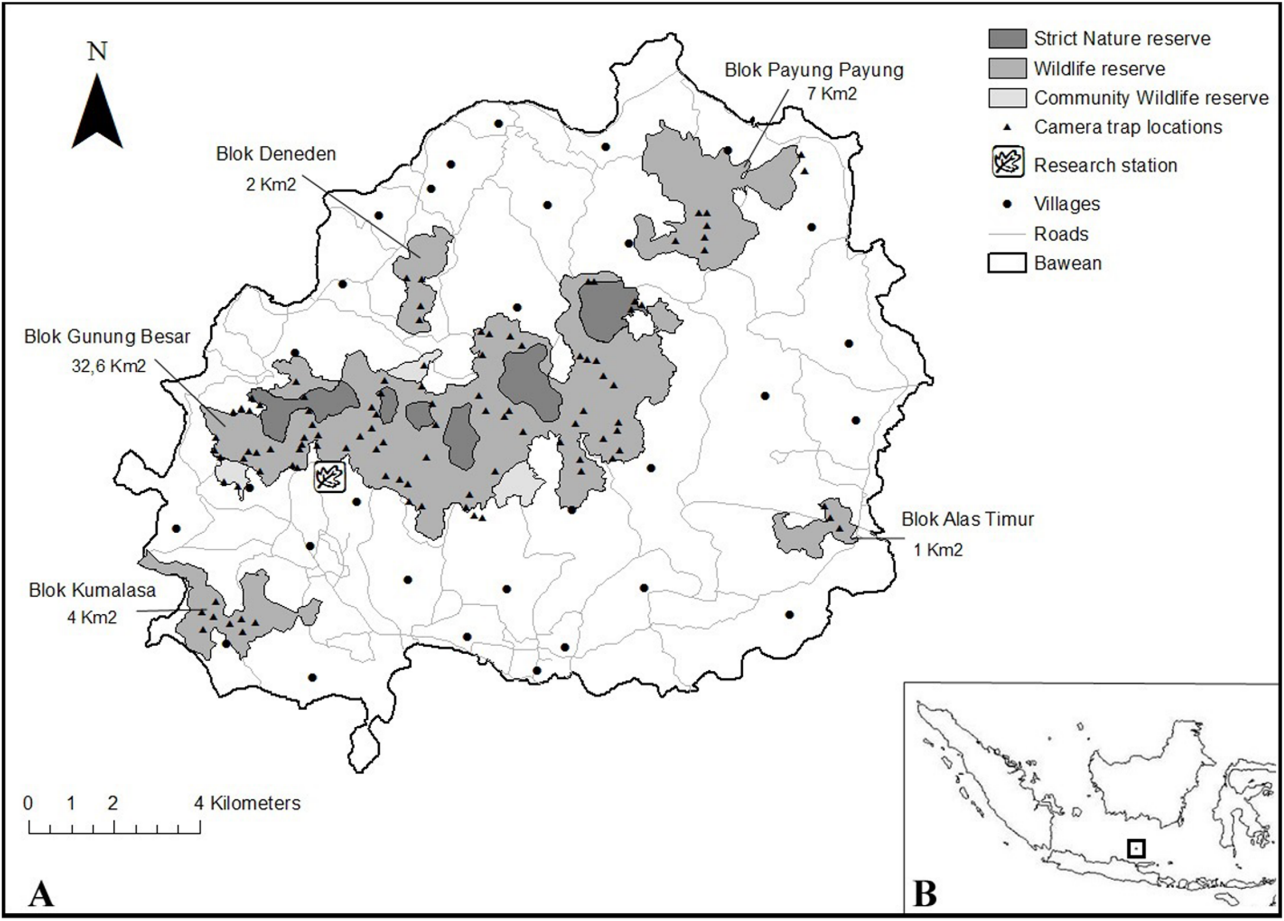
(**A**) Zonation of protected areas on Bawean Island, camera trap locations and the location of the research station. (**B)** Location of Bawean in the Java Sea. A buffer of 100 meter from the outer forest edge was included into the ArcGIS base map due to the uncertainty of the official zonation (see text).

The 192 km^2^ large Bawean island (Fig 1; S 05°46’0.00”, E 122°40’0.00”) is a remnant of an extinct volcanic mountain located in the Java Sea approximately 120 km north of Java and 260 km south of Kalimantan. The island used to be part of the larger Sundaland landmass that existed until the Late Miocene some 5 million years ago [15]. During the last glacial maximum (ca. 12,000 years ago), when sea levels were much lower, the island was connected to both the present land areas of Java and Borneo and the vegetation of Bawean appears to have been more open than today, with grassland and woodland savannahs dominating [16].

Topographically, the island presently consists of numerous small mountains, with the highest peak being Gunung Besar at 646 m asl [17]. The weather on the island is seasonal with heavy rains from December through March and a dry season from April through November [18]. The current human population is about 90,000, having grown relatively little from an estimated population of 44,000 inhabitants in the early 1900s [19]. Historically, the vegetation of this island likely consisted of a deciduous monsoon forest type [20], but people probably used and converted these forests extensively for hundreds of years. Remaining forests were protected in forest reserves in the 1930s [21], which were transformed into the Bawean Nature Reserve in 1995 [22]. The Bawean Nature Reserve roughly coincides with forest borders on the island and is subdivided into five wildlife reserves (38 km^2^), six strict nature reserves (7 km^2^) and three community wildlife reserves (1.6 km^2^). Despite these designations, small scale illegal logging and burning continue to occur in protected forests due to a lack of clear area boundaries and law enforcement ([17], Nur Syamsi, personal communication). Nijman [17] recognised five distinct habitat types on Bawean Island, four of which are covered by the protected areas (Table 1). This study was conducted in all of the protected areas on mainland Bawean.

**Table 1:** Distinct habitat types found at the study site. Adapted from Nijman [17].

Habitat type	Description
**Community forest**	Community owned forested gardens at the assumed borders of the protected areas consisting of a mixture of cultivated trees such as *Spondias pinnata*, *Artocarpus heterophyllus*, *Tectona grandis*, *Tamarindus indica*, *Bambussa* spp., *Arenga pinnata* and undergrowth dominated by either shrubs or grasses.
**Teak plantation**	Monoculture *T*. *grandis* stands inside the protected areas with undergrowth dominated by grasses and sparse herbaceous plant and shrub cover.
**Shrubland and degraded forest**	Patches inside the protected areas characterized by high, young (DBH<30cm) tree density and clear signs of logging and burning. Undergrowth is either dominated by a mix of grassland and herbaceous plants, or dense shrub cover. Tree species primarily represent those found in community forest, such as mixtures of *Bambussa* spp., *A*. *pinnata* and *T*. *grandis*.
**Tall forest**	Mature secondary or tertiary forest characterized by *Ficus variegata*, *F*.*s septica*, *Podocarpus rumphii*, and multiple *Eugenia* species, interspersed with dense patches of small trees.

### Data collection

Camera trapping took place from November 2014 until January 2015. For the estimation of population density, we randomly deployed 20 Cuddeback Long Range IR/E2 camera traps in five runs of seven days. We installed camera traps in the five protected forest areas Gunung Besar, Payung Payung, Kumalasa, Deneden and Alas Timur. We determined camera locations based on the following procedures. We superimposed a grid with 100 x 100m cells over the study area using the Fishnet and Clip tools in Arcmap10 [23]. This generated 3636 potential camera locations from which we randomly selected 100 camera locations in the field. We maintained a minimum between-point distance of 150 meters to prevent spatial autocorrelation between the camera traps [24]. We sampled the Gunung Besar (32 km^2^) forest area following this procedure from east to west in a total of four runs. Due to terrain difficulties, we sampled the areas Payung Payung (7 km^2^), Kumalasa (4 km^2^), Deneden (2 km^2^) and Alas Timur (1 km^2^) (see Fig 1) differently. We approximated the first randomly generated point as closely as possible, and placed a camera every 300 meters from the previous cameras in any direction that allowed this distance of travel by foot. To prevent oversampling of habitat features that species might actively seek out, e.g. roads and trails, we avoided sampling these features in the systematically selected locations [25]. Random locations can occasionally include roads or trails, but this will only be in proportion to their actual density in the landscape [26]. We set cameras at 30 seconds video mode with 1 minute intervals. Following Rowcliffe *et al*. [27] we measured the angle of detection, radial distance at detection and the distance travelled for the first three *S*. *blouchi* videos on each camera. These variables are required for computing the parameter estimates for the Rand Encounter Model (REM) analysis [27].

After installing the camera traps, we constructed a 10 x 10 meter plot with the camera at its centre, to provide a habitat assessment of the location. Firstly, we noted the area name and habitat type of the plot, the presence or absence of a wallow and GPS coordinates from which we could derive the altitude and distance to nearest border. Secondly, we measured tree density per m^2^ at each location using the T-square method [28] with two sample points. We measured the distance to the nearest tree from a random point in the plot. From this tree a perpendicular line was drawn and the distance to its nearest neighbour on the other side of the perpendicular line was measured. We took the nearest neighbouring tree as the starting point for a second measurement. Finally, we measured the average litter depth in cm in four 1 x 1m subplots in the corners of the plot.

### Analysis of Population Size

To estimate population density we defined the camera trap rate as the number of independent video events per 24 hour period. Individual animals pausing in front of the camera can trigger the camera multiple times resulting in non-independent observations [29]. Following Bowkett *et al*., [30] and Rovero *et al*., [29, 31], who focussed on a range of species, we aimed to reduce the bias from multiple detections of the same individual by setting a 1-hour independence interval for videos of individuals of the same sex and age class.

We derived standard descriptors of camera trap rate and population characteristics such as group size and sex ratio by using descriptive statistics with standard deviations. We computed sampling precision as the coefficient of variation (CV) of *S*. *blouchi* camera trap rates across cumulative camera days [31, 32]. Density estimation followed the equation provided by Rowcliffe *et al*., [25], with parameter estimators adjusted to be in line with later improvements to the model [27, 33].

$$D=\frac{y}{t}\frac{\pi }{vr(2+\theta )}g$$

In which D is the density obtained by multiplying the number of independent observations *y* per unit time *t*, with π divided by the product of parameter estimators day range *v*, effective radial distance *r* and effective detection angle *θ*. The outcome can then be multiplied by group size *g* for group animals [25]. We computed effective radial distance and detection angle in Distance 6.0 using a line-transect model for angle and a point-transect model for distances [27, 34]. Afterwards, we multiplied effective angle by 2 and converted to radians to obtain the full field of detection. Parameter *v*, day-range, was filled in as the average speed of movement of the pigs in front of the camera in m/second, extrapolated to km/day and multiplied by the proportion of time spent active. We obtained the proportion of time spent active by using the R package ‘activity’ [33] and used a Chi-square test to compare the amount of observations from sunset to sunrise and sunrise to sunset. Year-round data on time of sunrise and sunset were obtained from the Canadian National Research Council [35]. As camera traps might not be successful in capturing all individuals in a social group, the inclusion of group size in the model based on the average number of individuals in all videos might underestimate the actual number of individuals. However, basing group size on counts at wallows alone [36] can result in overestimating group size as multiple groups might migrate to the same wallow if water is scarce [37]. Therefore, we calculated the multiplier group size by merging the average group size from 35 independent videos at wallows and 92 without wallows. The density equation is filled in twice, once without the group size multiplier to provide a lower estimate of density and once with the group size multiplier to provide an upper estimate. We extrapolated the estimated densities per km^2^ to the total protected area size on Bawean to provide estimations of population size. We used a propagation of error approach, a calculus derived statistical calculation, to determine the effects of the uncertainty of the parameter variables on the uncertainty of the density function [38]. The uncertainty of the density estimation D is equal to the square root of the squared sums of the uncertainty of the parameters day range, radius and angle, multiplied by the partial derivative of these parameters [39].

$$\sigma D=\sqrt{\;{\left(\frac{\partial D}{\partial v}\partial v\right)}^{2}{\left(\frac{\partial D}{\partial r}\partial r\right)}^{2}{\left(\frac{\partial D}{\partial \theta }\partial \theta \right)}^{2}}$$

All statistical tests were performed in SPSS 20.0 and parameter estimates and standard errors were computed in Excel 2013.

### Analysis of Habitat preferences

#### Generalized Linear Models

We defined camera trap rate as the total number of independent videos at each plot [40]. We used an information theoretic approach for model selection and inference, based on the AICc and Akaike weight values in Generalized Linear Models (GzLM) to determine which of the six habitat variables habitat type, tree density, altitude, distance to nearest border, litter depth, and area had a significant effect on the camera trap rate [41]. As we detected overdispersion in the trap rate data, we chose a negative binomial GzLM type [42]. We included a natural logarithmic transformation of camera trap hours (ch) as an offset variable as we expected camera trap rate to increase with increasing camera hours.

$$\text{ln }ch=\frac{{\text{log}}_{10}ch}{{\text{log}}_{10\;}e}$$

We checked multicollinearity using collinearity diagnostics with a threshold at a Variance Inflaction Factor (VIF) lower than 2.5. Although sample size of camera traps at wallows was too small to be included into the GzLM (n = 4), wallows may have important social and thermoregulatory functions in pig communities [43–45]. Therefore, we analysed the potential relationship of the number of observations between locations with and without a wallow separately using a Kruskall-Wallis One-way ANOVA. Next to this, we excluded the two smaller areas Deneden and Alas Timur as they only contained 4 and 3 camera trap rate values respectively. We ran multiple GzLMs covering all combinations for the remaining six habitat variables (n = 63). If there were multiple models competing for the first ranking model (e.g. Delta AIC<2), we computed a weighted average of the estimates for each variable across all the models in which that given variable was included instead of relying on the estimates of the best model (i.e. lowest AICc value). Following Grueber *et al*., [46], more complex models, i.e. a model containing variables 1,2,3,4, but with a lower AICc value than a less complex model with the same set of variables, e.g. variables 1,2,3, were excluded from the weighted average. A significant effect was obtained if the confidence intervals for the weighted averages excluded zero [47].

#### Occupancy

As a comparative method to the habitat preferences obtained through GzLMs we estimated how habitat predicts the occupancy of pigs with single season occupancy modelling using the R-package ‘unmarked’ [48, 49]. Models with all combinations of six habitat covariates were fit and ranked using AIC [41]. We removed records that were incomplete due to missing covariate values. Coefficients from all models within 2 AIC of the top model were averaged using the r function fitList in unmarked [48] to assess the relationship between occupancy and habitat characteristics. We calculated model averaged upper and lower confidence intervals for each covariate [41]. We estimated the total number of sites occupied as the sum of the mean of the posterior distribution of occupancy at each site [48]. We estimated model fit by computing the Mackenzie and Bailey goodness-of-fit Chi-squared test on the global model.

## Results

### Species identification

Based on the videos obtained we determined that only one species of wild pig occurs on Bawean, i.e., *S*. *blouchi*. This was determined for males by the presence of warts and large tufts of golden yellowish hair fanning out from both sides of the head [50]. The females and young of the two species are more difficult to distinguish [9, 51]. However, all individuals possessed a clear contrasting band of golden yellowish hair running alongside the abdomen, characteristic of *S*. *verrucosus* [50], whereas pelage colouration in *S*. *s*. *vitattus* is a uniformly grizzled black with tips of yellow or red, and no light ventral band [52, 53].

### Population estimation

**Fig 2:**
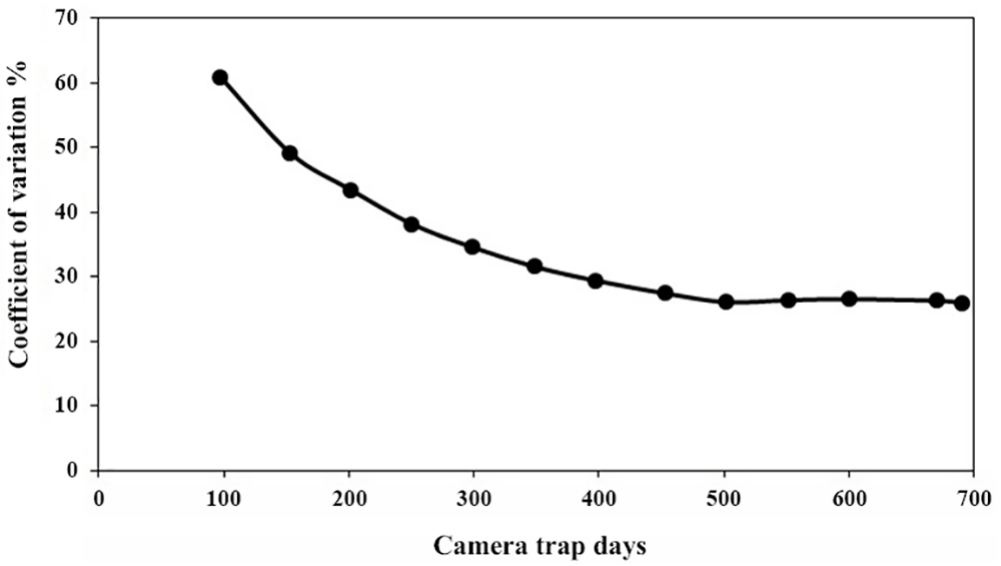
Camera trapping sampling precision for *S*. *blouchi*. Sampling precision was expressed as CV of *S*. *blouchi* camera trap rate with cumulative sampling effort (number of camera trap days) in the protected areas.

The total number of camera days was 690.31, with 92 independent video events. Overall we obtained *S*. *blouchi* videos at 45 of the 105 sampled locations. The variation in camera trap rate stabilized at a trapping effort of approximately 500 camera days (Fig 2), indicating that satisfactory sampling precision was reached. Mean camera trap rate was 0.12 ± 0.20 SD with a total of 162 individuals recorded.

**Fig 3:**
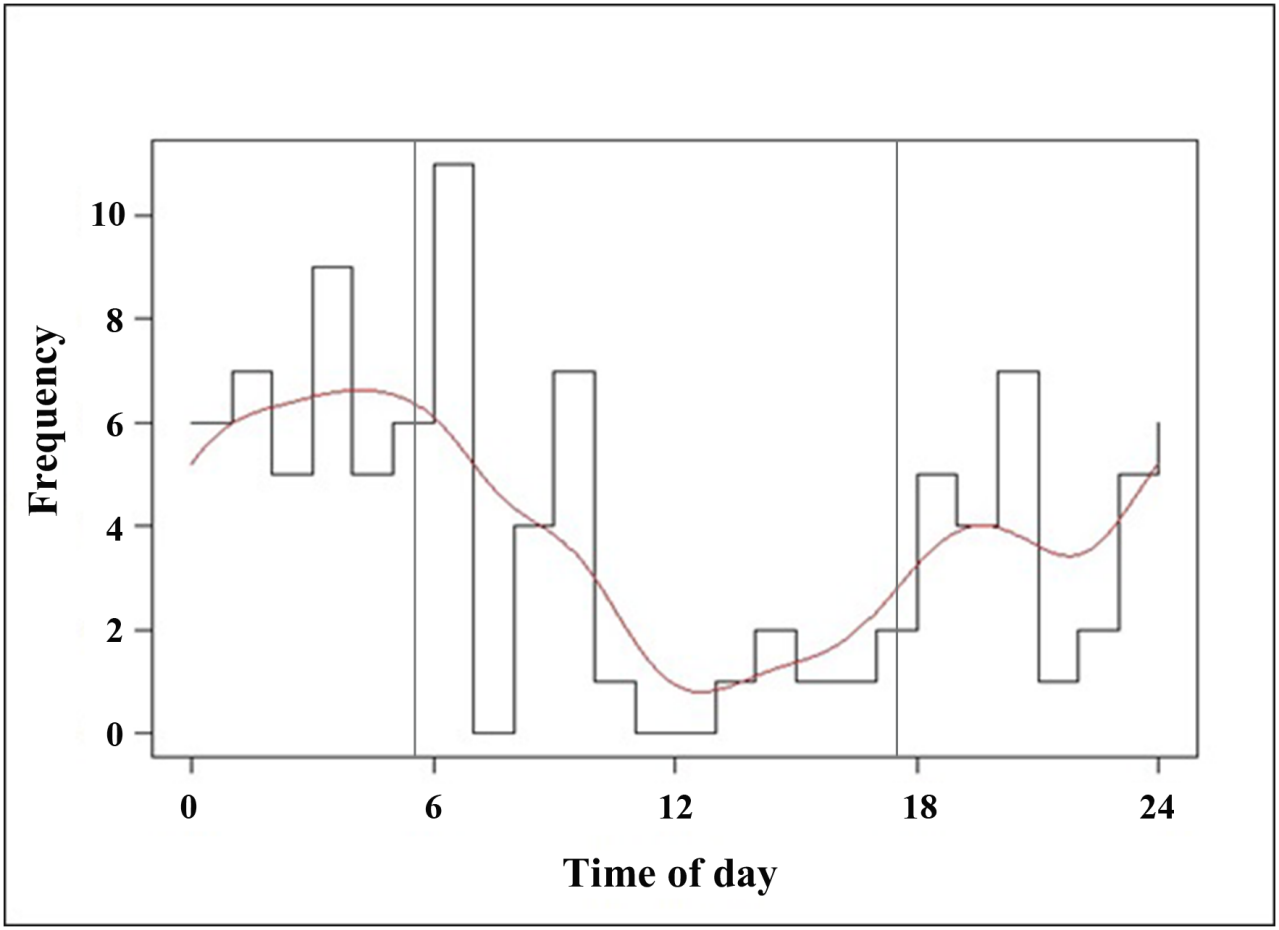
Proportion of observations per time of day spent active fitted with a line function using the activity package in R [33] to determine proportion of time spent active. Vertical lines indicate sunrise and sunset at approximately 05.30 and 17.50 year-round.

Adult male to adult female sex ratio was 1:2 (adult males n = 35, adult females n = 67, juveniles n = 30, unknown n = 30) and mean group size in videos was 2.18 (mean at wallows = 2.60 ± 1.35 SD; mean at locations without wallows = 1.76 ± 1.36 SD). The proportion of time spent active per day was found to be 0.58 with high proportions of observations during the night and peaks in the early morning and late afternoon (Fig 3). Activity was significantly higher between sunset and sunrise than between sunrise and sunset (Chi-square χ^2^ = 27.409, Df = 1, *P*<0.001).

Based on the included parameter estimates (Table 2) in the REM, we obtained a lower and upper density estimate of 3.7 ± 0.9 SE and 8.1 ± 0.9 SE individuals per km^2^. This, if extrapolated to total protected area size, amounts to an estimated population size of between 172 and 377 individuals for Bawean, assuming that no pigs are permanently resident outside of the protected areas.

**Table 2:** Parameter estimates for *S*. *blouchi* included in the REM.

Parameter estimates	Mean	S.E.	N. videos
Trap rate (*y/t*)	0.1374	0.0338	92
Day range (*v*, km day)	9.7802	3.7170	57
Radial distance (*r*, km)	0.0039	0.0003	63
Angle (radians, θ)	0.3920	0.0330	62
Group size (*g*)	2.18		57

### Habitat preferences

#### Generalized Linear Models

**Fig 4:**
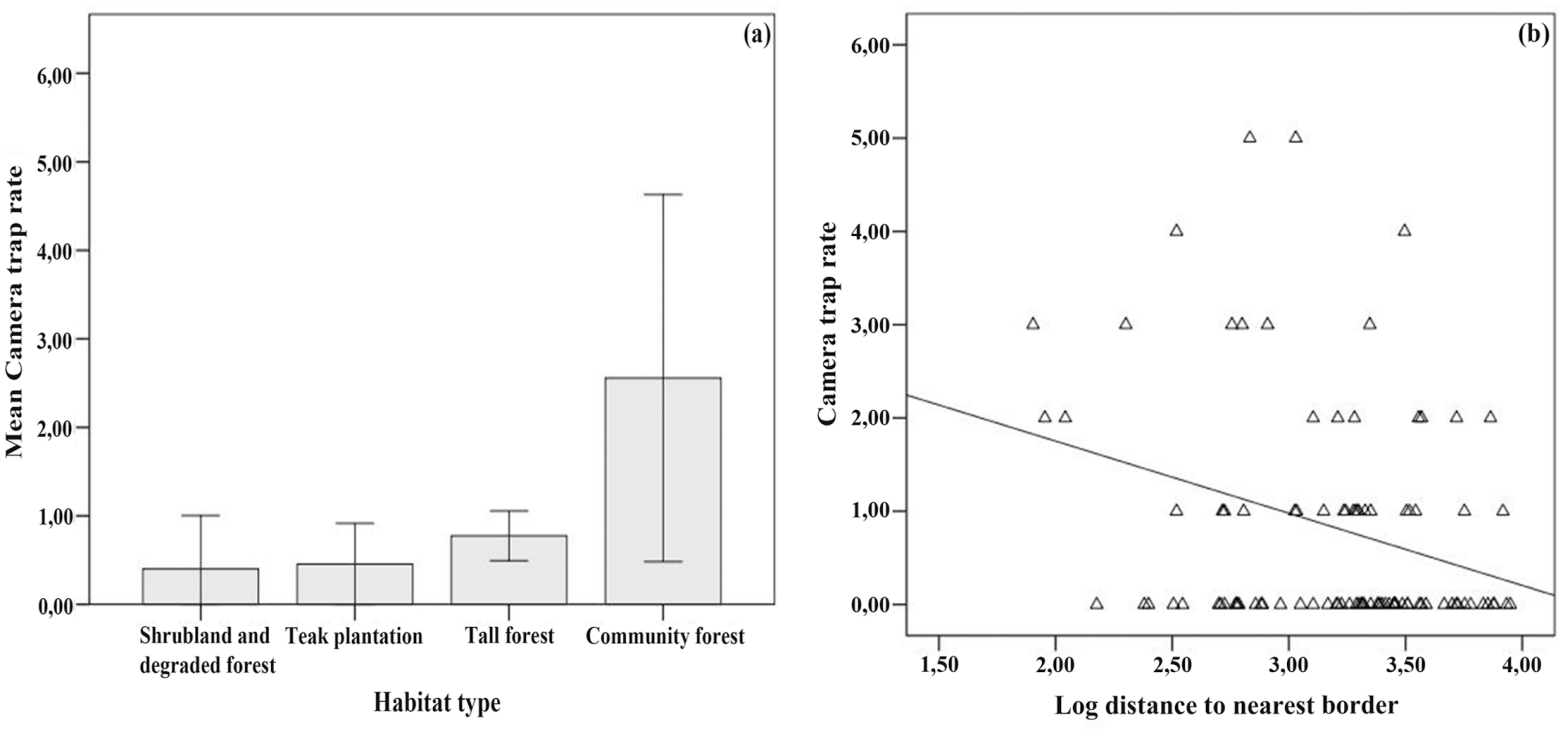
(a) Mean camera trap rate across the different habitat types shrubland and degraded forest (0.40 ± 0.26), teak plantation (0.45 ± 0.20), tall forest (0.77 ± 0.14) and community forest (2.55 ± 0.89), (b) Camera trap rate as a function of distance to nearest border (R^2^ = 0.067).

We found a total of thirteen competing models for the first ranking model (i.e. Delta AICc<2; Table 3; Table 4). We did not find any multicollinearity between the covariables used in the models. Based on the confidence intervals computed from the weighted averages of the habitat variables, we found two variables to have a discernible effect on camera trap rates (Fig 4a and 4b). Camera trap rate was higher in community forest compared to shrubland and degraded forest (95% CI = -0.042, -0.502), teak plantations (95% CI = -0.033, -0.489) and tall forest (95% CI = -0.009, -0.330). Next to this, the camera trap rate was discernibly lower with increasing distance to the nearest border (95% CI = -0.0318, -0.524). Area (95% CI = 0.537, -0.191; 0.619, -0.271), Tree density (95% CI = 0.262, -0.037), altitude (95% CI = 0.290, -0.314) and litter depth (95% CI = 0.0143, -0.052) were not found to have a discernible effect on trap rates. Camera trap rate was significantly higher at locations that had a wallow than locations were no wallow was present (Kruskal-Wallis, *df* = 1, H = 11.262, *P*<0.001).

**Table 3:** Top 13 AICc models competing for first ranking model. Beta coefficients ± standard errors for each covariate are listed. The presence of categorical variables Habitat type and Area in the models is indicated with the • symbol.

Model ID	Habitat type	Tree density	Altititude	Dist. to near. border	Litter depth	Area	No. of param-eters	AICc	Delta AICc	Akaike weight
1	•	0.676 ± 0.459		-0.842 ± 0.405			7	241.1	0.0	0.0875
2		0.641 ± 0.421		-1.130 ± 0.358			3	241.2	0.1	0.0835
3	•	0.652 ± 0.468	0.188 ± 0.795	-0.837 ± 0.423			8	241.5	0.4	0.0726
4	•	0.597 ± 0.466		-0.835 ± 0.421		•	10	241.8	0.6	0.0640
5	•	0.848 ± 0.484		-0.836 ± 0.406	-1.124 ± 0.905		8	241.9	0.7	0.0604
6		0.606 ± 0.439	-0.461 ± 0.827	-0.997 ± 0.386		•	7	241.9	0.8	0.0572
7		0.547 ± 0.428		-1.106 ± 0.363		•	6	242.0	0.9	0.0560
8		0.787 ± 0.462		-1.118 ± 0.364	-1.220 ± 0.838	•	7	242.1	1.0	0.0522
9	•	0.803 ± 0.488	-0.456 ± 0.834	-0.878 ± 0.426			9	242.2	1.1	0.0504
10	•	0.620 ± 0.473	-0.238 ± 0.845	-0.762 ± 0.443		•	11	242.2	1.1	0.0503
11		0.787 ± 0.465	-0.103 ± 0.880	-1.073 ± 0.394	-1.150 ± 0.898	•	8	242.6	1.5	0.0403
12			-0.168 ± 0.805	-0.894 ± 0.375		•	6	242.8	1.7	0.0365
13	•	0.756 ± 0.492		-0.840 ± 0.422	-1.009 ± 0.912	•	11	242.9	1.8	0.0349

**Table 4:** Beta coefficients ± standard errors for the categorical) in the top 13 AICc models competing for first ranking model. Variables Habitat type (1 = Shrubland & Degraded forest; 2 = Teak stands; 3 = Tall forest; 4 = Community forest, reference category, not included) and Area (1 = Gunung besar; 2 = Payung-payung; 3 = Kumalasa, reference category, not included.

Model ID	Habitat type 1	Habitat type 2	Habitat type 3	Area 1	Area 2	No. of param-eters	AICc	Delta AICc	Akaike weight
1	-2.058 ± 0.766	-1.360 ± 0.746	-1.178 ± 0.509			7	241.1	0.0	0.0875
2						3	241.2	0.1	0.0835
3	-2.068 ± 0.768	-1.372 ± 0.746	-1.178 ± 0.511			8	241.5	0.4	0.0726
4	-1.784 ± 0.796	-1.160 ± 0.806	-1.147 ± 0.519	0.530 ± 0.707	0.702 ± 0.872	10	241.8	0.6	0.0640
5	-1.979 ± 0.771	-1.513 ± 0.762	-1.088 ± 0.516			8	241.9	0.7	0.0604
6				0.832 ± 0.727	0.767 ± 0.901	7	241.9	0.8	0.0572
7				0.704 ± 0.705	0.603 ± 0.861	6	242.0	0.9	0.0560
8				0.714 ± 0.707	0.479 ± 0.867	7	242.1	1.0	0.0522
9	-1.997 ± 0.774	-1.538 ± 0.768	-1.095 ± 0.517			9	242.2	1.1	0.0504
10	-1.758 ± 0.796	-1.179 ± 0.806	-1.119 ± 0.521	0.613 ± 0.731	0.799 ± 0.915	11	242.2	1.1	0.0503
11				0.762 ± 0.731	0.531 ± 0.922	8	242.6	1.5	0.0403
12				0.761 ± 0.724	0.661 ± 0.896	6	242.8	1.7	0.0365
13	-1.694 ± 0.802	-1.292 ± 0.821	-1.049 ± 0.528	0.550 ± 0.709	0.632 ± 0.876	11	242.9	1.8	0.0349

#### Occupancy

**Fig 5:**
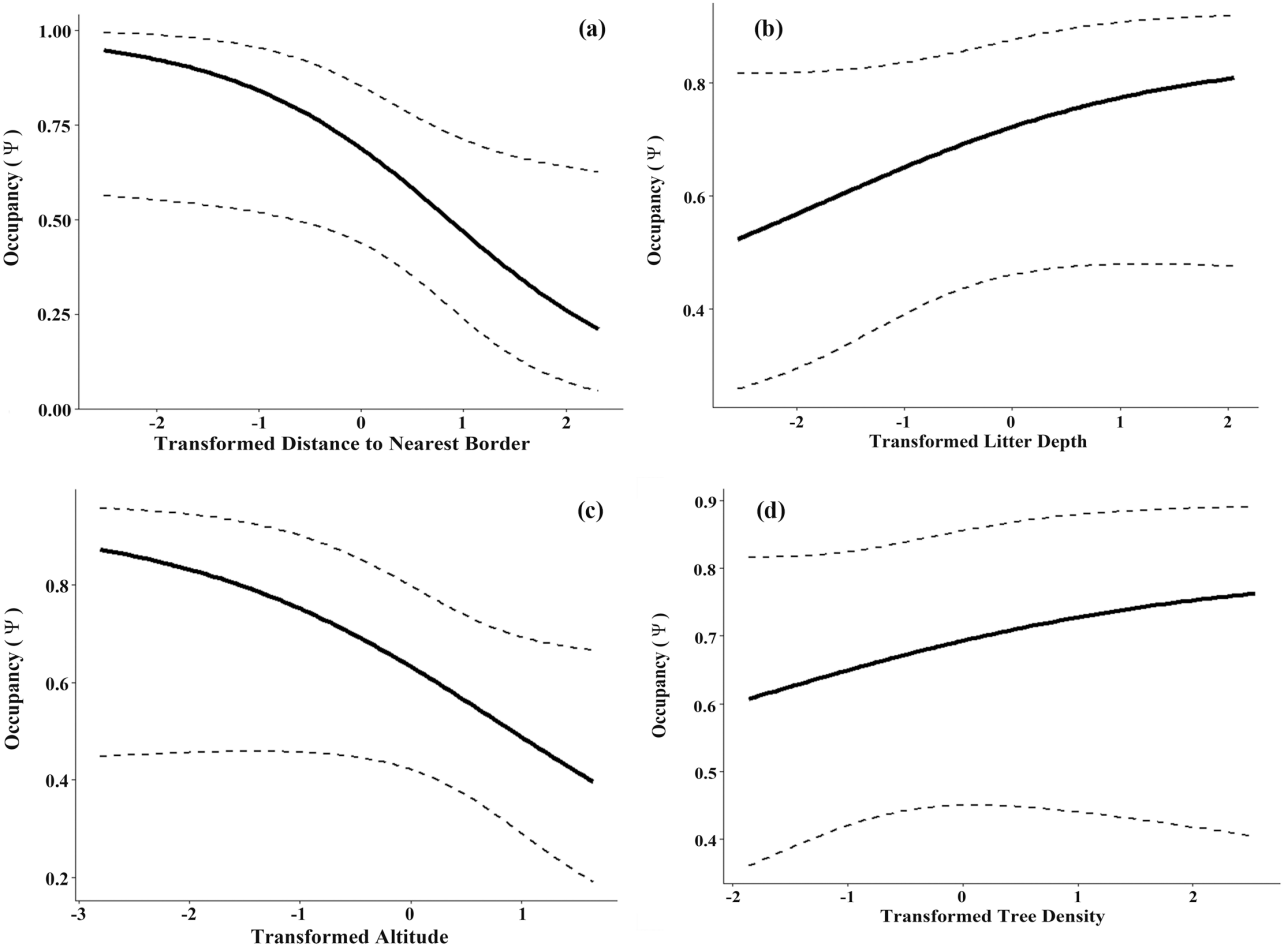
Model-averaged predicted occupancy (Ψ) in relation to (a) The distance from the nearest border (95% CI = 0.194. -1.040), (b) Litter depth (95% CI = 0.454, -0.146), (c) Altitude (95% CI = 0.179, -0.716) and (d) Tree density (95% CI = 0.304, -0.121). Predictor variables were log transformed.

Twelve camera sites were removed from the data due to missing covariate information. Of the remaining 90 sites, wild pigs were detected at 43, giving a naïve occupancy of 0.48. Accounting for detection error using occupancy models corrected this estimate upward. Model-averaged detectability was 0.432 (95% CI = 0.330, 0.540). The sum of the means of the site posterior distributions of occupancy estimated by the top ranking model was 52, increasing the estimated proportion of sites occupied to 0.58. We observed no evidence of lack of model fit from the global model (*X*^2^ = 7.66, p = 0.33, $\overset{⌢}{c}$ = 1.13). The five models ranked within 2 AIC points of the top ranking model included all variables with distance to nearest border appearring in all five (Table 5). However, model averaged confidence intervals for all variables crossed zero (Table 6). The probability of occupancy increased with both litter depth and tree density, but decreased with distance to nearest border and altitude (Fig 5).

**Table 5:** Top 5 competing AIC models competing for first rank. Beta coefficients ± standard errors for each covariate are listed.

Model ID	Habitat type	Tree density	Altitude	Distance to nearest border	Litter depth	Area	No. of param-eters	AIC	Delta AIC	Akaike weight
1			-0.711 ± 0.389	-1.004 ± 0.493	0.653 ± 0.403		4	289.8	0.0	0.1329
2		0.490 ± 0.442	-0.896 ± 0.493	-1.284 ± 0.629	0.631 ± 0.420		5	290.2	0.4	0.1067
3		0.520 ± 0.414	-0.621 ± 0.392	-0.880 ± 0.442			4	241.5	1.1	0.0750
4			-0.428 ± 0.298	-0.604 ± 0.313			3	241.8	1.1	0.0745
5				-0.656 ± 0.318			2	241.9	1.1	0.0635

**Table 6:** Averaged Beta values and upper and lower 95% confidence intervals for occupancy covariates.

Covariates	Tree density	Upper 95% CI	Lower 95% CI
Tree density	0.091	0.304	-0.121
Altitude	-0.269	0.179	-0.716
Dist. nearest border	-0.423	0.194	-1.040
Litter depth	0.154	0.454	-0.146

## Discussion

### Population characteristics

Our density estimate for *S*. *blouchi* of 3.7–8.1 pigs/km^2^ seems to be low compared to other pig species in SE Asia. For example, densities of *S*. *celebensis* on Sulawesi were estimated at 1230 pigs/km^2^ [54–56], while research by Pauwels [57], Ickes [58] and Yong *et al*., [59], estimated *S*. *scrofa* densities at 27–47 pigs/km^2^ in seasonal tropical forest sites in Java, Malaysia and Singapore. No density estimates exist for the close relative of *S*. *blouchi*, *S*. *verrucosus* on Java. Pig densities are likely to fluctuate significantly with resource availability, a pattern taken to the extreme by the bearded pig *S*. *barbatus* with models suggesting tenfold increases in abundance in response to consecutive masting events in dipterocarp forests [60, 61]. Bawean’s ecological conditions are characterised by strong seasonality, an unquantified but consistent hunting pressure [Nurs Syamsi, personal communication], and an absence of natural predators. Multi-annual research might give an indication of strong fluctuations in population numbers.

The 1:2 male to female sex ratio found in this study for *S*. *blouchi* seems to fit the variable range of sex ratios observed in wild suidae throughout the world. A 1:2 sex ratio has been observed in forest hogs (*Hylochoerus meinertzhageni*) [62] and a ratio of 1:1.7 was observed in the Sulawesi warty pig (*S*. *celebensis*) [63]. Wild boar (*S*. *scrofa*) and bush pigs (*Potamochoerus porcus*), though, both have a sex ratio close to 1:1 [64, 65].

We found a mainly nocturnal activity pattern for *S*. *blouchi* with peaks in the early morning and late afternoon. A similar activity pattern has been observed in feral pigs (*S*. *scrofa domesticus*) [66] and *S*. *scrofa* [50].

We note that the minimum number of 50 independent observations required for REM analysis, suggested in Rovero *et al*., [67], was reached after 343 camera days. However the CV of camera trap rate only started to level off after 500 camera days. This indicates that a higher number of camera days may be required for a reliable density estimate than what is required for obtaining the minimum number of observations alone.

### Habitat preferences

We used both GzLMs and occupancy modelling to investigate habitat preferences. GzLMs showed a significantly higher *S*. *blouchi* trap rate in community forests compared to the other habitats and a significant negative relationship between *S*. *blouchi* trap rate and the distance to nearest border. Occupancy modelling corroborated our results from the GzLMs, which demonstrated a positive association between pig habitat use and areas near borders. In addition, occupancy models indicated that pig use is positively associated with tree density and litter depth. Whereas the associations of these variables were not significant based on model averaging, their inclusion in the top ranking models and especially the presence of distance to nearest border in all top models, indicates they should be included when predicting the area of Bawean used by warty pigs.

Overall *S*. *blouchi* can be regarded as a habitat generalist with a preference for semi-open cultivated habitat, which is similar to *S*. *verrucosus* [68, 10, 11]. During the day the species was observed only in the tall forest and based on the low number of daytime videos is presumed to be mainly inactive during day-light hours [33]. The most likely explanation for the habitat preference of community forests is the availability of more energy-rich foods e.g. roots and tubers, in these cultivated areas compared to other habitats [69]. *S*. *blouchi* might differ in this respect from *S*. *verrucosus* on Java, for which teak stands have been suggested to be a preferred habitat [9, 11]. We interpret the negative relationship between *S*. *blouchi* density and distance to nearest border as a direct link with increasing distance to the community forests at the edge of the forest. However, during this study we frequently observed signs of *S*. *blouchi* rooting in the teak stands. Finally, we found a potential effect of wallow presence on warty pig density, as is expected based on the important roles these habitat features play in thermoregulation and social interactions [45].

### Conservation Recommendations

Our findings provide a basis for conducting a Red List status assessment for *S*. *blouchi*, with the new data allowing the determination of the number of subpopulations, the number of mature individuals, population size and estimated population trend, extent of occurrence and area of occupancy. On behalf of the IUCN/SSC Wild Pig Specialist Group we conducted this assessment and concluded that the species should be listed as Endangered, primarily because of a population size estimated to number fewer than 250 mature individuals. This information may further assist in effective future conservation planning for *S*. *blouchi*.
